# Inhibition of TRAF6 improves hyperlipidemic acute pancreatitis by alleviating pyroptosis in vitro and in vivo rat models

**DOI:** 10.1186/s13062-023-00380-y

**Published:** 2023-05-10

**Authors:** Biwei Wei, Zhou Su, Huiying Yang, Yong Feng, Chunmei Meng, Zhihai Liang

**Affiliations:** 1 Department of Gastroenterology, The First Affiliated Hospital of Guangxi Medical University, No. 6 Shuangyong Road, 530021 Nanning, Guangxi Zhuang Autonomous Region China; 2 Life Sciences Institute, Guangxi Medical University, Nanning, China

**Keywords:** Hyperlipidemic acute pancreatitis, Tumor necrosis factor receptor-related factor 6, Pyroptosis, Cysteinyl aspartate specific proteinase-1, Gasdermin D

## Abstract

**Objective:**

Hypertriglyceridemia (HTG) is one of the common causes of acute pancreatitis (AP). Hyperlipidemic acute pancreatitis (HTG-AP) is associated with higher mortality owing to its tendency for greater severity and rapid progression. The purpose of this study was to explore the mechanism of involvement of tumor necrosis factor receptor-related factor 6 (TRAF6) in pyroptosis during HTG-AP.

**Methods:**

The HTG environment was simulated with palmitic acid treatment in vitro and a high-fat diet in vivo. Cerulein was used to establish the HTG-AP model, followed by genetic and pharmacological inhibition of TRAF6. Pyroptosis activation, inflammatory reaction, and the interaction between TRAF6 and pyroptosis in HTG-AP were assessed.

**Results:**

HTG was found to aggravate the development of pancreatitis, accompanied by increased pyroptosis and enhanced inflammatory response in HTG-AP models. Mechanistically, TRAF6 downregulation decreased the activation of pyroptosis in cerulein-induced HTG-AP.

**Conclusion:**

Collectively, inhibition of TRAF6 improved HTG-AP and the associated inflammation by alleviating pyroptosis.

## Introduction

Acute pancreatitis (AP) is a common inflammatory disease of the pancreas characterized by acute epigastric pain, nausea, vomiting, and elevated serum amylase level [1]. Owing to the changes in lifestyle and dietary structure, hypertriglyceridemia (HTG) has become the second major cause of AP in China [2]. Moreover, HTG is the third and fourth common cause of AP in Europe and the United States, respectively (after gallstones, alcohol or/and post-ERCP) [3]. Hyperlipidemic acute pancreatitis (HTG-AP) accounts for approximately 5% of all cases of AP [4, 5]. Persistent multiple organ failure in the early stage and infectious necrosis in the late stage are associated with poor prognosis and mortality in HTG-AP [6]. However, the pathogenesis of HTG-AP is not well elucidated. Genetic, metabolic, environmental, and/or patient-specific factors may be involved in the causation of this disease [7].

Several forms of cell death may be involved in the early course of AP [8]. Previous studies have suggested that apoptosis and necrosis are the two main modes of cell death in pancreatic acinar cells. AP is associated with extensive apoptosis of pancreatic acinar cells, while SAP is associated with necrosis of pancreatic acinar cells. Apoptosis is a rigorously regulated process involving a variety of cysteinyl aspartate specific proteinase (caspase). The cell membrane structure is intact during apoptosis so that cell contents are not released, and the inflammatory reaction is mild. Necrosis is a process that activates the receptor-interacting protein 1 (RIP1)/ RIP3/mixed lineage kinase domain-like (MLKL) pathway. Phosphorylated MLKL accumulates in the cell membrane to form pores, resulting in cell lysis and release of its contents to promote inflammatory responses [9–12]. However, in a study, 40% of pancreatic acinar cells were found to have died even after inhibition of necroptosis [13]. Recently, several studies have suggested the involvement of pyroptosis (another form of cell death) in pancreatic necrosis and HTG-AP-related systemic inflammation. The injured pancreatic acinar cells were found to exhibit the characteristics of pyroptosis and participate in HTG-AP [14]. The actual difference between pyroptosis, apoptosis and necroptosis depends on the involvement of different caspases. Caspase-1-mediated pyroptosis is known as the classical pathway of pyroptosis [15]. In the classical pathway, pattern recognition receptors recognize upstream signals and assemble apoptosis-associated speck-like proteins to activate inflammasomes. The inflammasomes then recruit caspase-1 precursors for cleavage into active caspase-1. Activated caspase-1 can cleave gasdermin D (GSDMD) to form the N-terminal domain with hole-punching function leading to the activation of pyroptosis. At the same time, it promotes the processing and release of IL-1β and IL-18, expanding the inflammatory response [16, 17].

Tumor necrosis factor receptor-related factor 6 (TRAF6), a family member of TRAF, is involved in a variety of acute inflammatory diseases via various signaling pathways [18–20]. TRAF6 is a central adaptor protein of upstream toll-like receptors (TLRs) and downstream nucleotide-binding oligomerization domain-like receptor family pyrin domain containing 3 (NLRP3) inflammasome that mediates myeloid differentiation factor 88 (MyD88), recruits interleukin-1 receptor-associated kinase-1 (IRAK-1) and IRAK4 forming a complex [21, 22]. Moreover, NLRP3 was shown to trigger caspase-1-dependent pyroptosis in pancreatic acinar cell injury [23]. Hence, we conducted a series of experiments to determine the function and underlying mechanism of participation of TRAF6 in pyroptosis during AP. In our previous studies, we found high expression of TRAF6 in cerulein-induced AP in vivo, and overexpression of *TRAF6* gene was found to activate pyroptosis to induce AP in vitro [24]. Therefore, we hypothesized that TRAF6 may mediate cerulein-induced HTG-AP by regulating pyroptosis. The present study aimed to further investigate the mechanism of TRAF6 in pyroptosis of HTG-AP by genetic and pharmacological inhibition of TRAF6 using in vivo and in vitro models.

## Materials and methods

### Cell culture and transfection

Rat pancreatic acinar AR42J cells were obtained from the American Type Culture Collection (ATCC, USA) and cultured in Minimum Essential Medium (MEM, Hyclone, Logan, UT, USA) supplemented with 10% fetal bovine serum (FBS, Hyclone). According to the manufacturer’s instructions, pancreatic acinar cells were transfected with TRAF6 siRNA and control siRNA using Lipofectamine 2000 (Invitrogen, Carlsbad, CA), and control siRNA as a negative control. The optimal concentrations of 0.5 mmol/L palmitic acid and 5 nM cerulein (Sigma-Aldrich, St Louis, MO, USA) intervention were selected for the next experiments. The TRAF6 siRNA sequence used for transfection is shown in Table 1.

**Table 1:** The sequence of siRNAs targeting TRAF6

Name	Sequence 5′-3′
si-NC	UUCUCCGAACGUGUCACGUTT
si-TRAF6	GGUAAAGUGUCCAAAUAAAGG

**Table 2:** Primer sequences used in this study

Gene name	Forward Primer	Reverse Primer
TLR9	TGTTGCCTTTACTGCAGCATCTC	CTCTGCGCCTTATCGAACACC
TRAF6	TTTGGCGTCGGAGACACTTG	TCGCTTGAAGACTGGCTGGA
NLRP3	CTGAAGCATCTGCTCTGCAACC	AACCAATGCGAGATCCTGACAAC
caspase-1	ACTCGTACACGTCTTGCCCTCA	CTGGGCAGGCAGCAAATTC
GSDMD	GAAACTCTCAAGCTCATGGTTCTGG	CGCAGCATACACACATTCATGG
β-actin	TTGCTGACAGGATGCAGAA	ACCAATCCACACAGAGTACTT

### Animals

Four-week-old male Sprague-Dawley rats were obtained from the Experimental Animal Center of the Guangxi Medical University (Nanning, China). Rats were housed in a controlled environment (12 h light/dark cycle, 26˚C temperature, 60% humidity) with *ad libitum* access to standard laboratory chow and water. The animal experimental protocols were approved by the Institutional Animal Care and Use Committee of Guangxi Medical University (No.201910036).

### HTG-AP rat model establishment and treatment

Rats were randomly divided into normal groups and HTG groups, fed with normal diet and high-fat diet (82.8% normal diet, 15% lard, 2% cholesterol, 0.2% sodium cholate, Solarbio, Beijing, China) for 4 weeks, respectively. Blood samples were collected from retroorbital veins to determine the HTG model by measuring serum TG levels. AP and HTG-AP were induced by seven intraperitoneal injections (1 h interval) of cerulein (dose: 50 µg/kg); control rats received intraperitoneal injections of the same dose of normal saline. Regarding the treatment groups for AP and HTG-AP, an inhibitor of TRAF6, MG-132 (within DMSO vehicle, Selleck Chemicals, Houston, TX, USA) was intraperitoneally injected for intervention 30 min before modeling. All rats were sacrificed at 24 and 48 h after the last cerulein or saline injection.

### Histopathological examination and serum amylase level

Pancreatic histopathologic examination and serum amylase determination were performed as previously described [25].

### CCK8 assay

Cells were added into a 96-well culture plate and incubated at 37 °C. Then the cells were collected at each time-point (0 h, 24 h, 48 h, 72 h) and subjected to cellTiter 96 one solution cell proliferation assay (Promega, Madison, WI, USA). The absorbance of each sample was measured at 490 nm wavelength after incubation for 4 h.

### ELISA

The levels of IL-1β and IL-18 in cell supernatant (ab100767, ab213909, all from Abcam) and rat serum (BMS630, Invitrogen) were determined using an enzyme-linked immunosorbent (ELISA) assay kit, according to the manufacturer’s instructions.

### Western blot analysis

Pancreatic acinar cells and fresh pancreatic tissues were lysed in RIPA lysis buffer with 1% phenylmethylsulfonyl fluoride (PMSF) to extract total proteins, which were determined using a Bicinchoninic Acid (BCA) kit (Solarbio). 10% and 15% SDS-polyacrylamide gels (Bio-rad, Hercules, CA, USA) were used to isolate the same mass of proteins, which were subsequently transferred onto polyvinylidene fluoride membranes (PDVF). The membranes were blocked using 5% skimmed milk for 45 min at room temperature to inhibit nonspecific binding, and washed three times for 10 min in Tris-buffered saline Tween (TBST) (containing 0.5 mM Tween-20). Then the membranes were incubated overnight at 4 °C with the following primary antibodies: anti-TLR9 (sc-52,966, 1:200, Santa Cruz Biotechnology, Santa Cruz, CA, USA); TRAF6 (ab33915, 1:1000, Abcam, Cambridge, UK); NLRP3 (NBP2-12446, 1:400, Novus Biologicals, CO, USA); caspase-1 (ab179515, 1:1000, Abcam); GSDMD (#39754S, 1:1000, Cell Signaling Technology, Boston, MA, USA); and GAPDH (ab181602, 1:10000, Abcam). After washing with TBST, the membranes were incubated with the corresponding secondary anti-rabbit/mouse IgG antibody (#4413, 1:10000, Cell Signaling Technology; SA5-35521, 1:10000, Invitrogen) for 1 h at room temperature. Protein bands were visualized by Odyssey Fc Imaging System.

### RNA isolation and quantification

Total RNA was extracted from pancreatic acinar cells and fresh pancreatic tissues using TRIzol reagent (Invitrogen) followed by determination of concentration and purity. Reverse transcription kits (RR047A, Takara, Japan) were used to obtain cDNA, and then RT-qPCR was performed with an Applied Biosystems 7500 Real-Time PCR System using the TB Green PCR kit (RR820, Takara). The mRNA expressions were quantified using the 2^−ΔΔCt^ method. The primer sequences are shown in Supplementary Table 2.

### Immunohistochemistry staining

Pancreatic tissue Sect. (4 μm) were baked, dewaxed, rehydrated, and their endogenous peroxidase activity was blocked. The sections were placed into sodium citrate buffer and heated in a microwave oven for 20 min. Subsequently, the sections were preincubated with normal goat solution for 10 min at room temperature, followed by overnight incubation with anti-GSDMD (Cell Signaling Technology) primary antibodies at 4 °C. On the next day, the sections were incubated with secondary antibody for 1 h at room temperature. After staining with diaminobenzidine and hematoxylin, at least five randomly selected images of each section were observed under a microscope.

### Immunofluorescent staining

Pancreatic acinar cells and pancreatic tissue sections were subjected to immunofluorescence staining. After fixation and dewaxing, respectively, cells and sections were infiltrated in sealing permeable liquid containing Triton X-100 for 30 min and washed three times for 5 min in PBS. The cells and sections were then incubated overnight at 4 °C with the following primary antibodies: anti-caspase-1 (Abcam) and GSDMD (Cell Signaling Technology). Finally, the cells and sections were washed and incubated with the corresponding fluorescence-conjugated second antibodies for 1 h at room temperature, followed by nuclear staining with DAPI for 2 min. A fluorescence microscope was used to capture randomly stained images of each sample.

### Transmission electron microscopy

Pancreatic acinar cells and fresh pancreatic tissues were fixed in electron microscope fixation fluid for 4 h at 4 °C. Cells and tissues were washed in PBS for 15 min, three times in total, and post-fixed in 1% osmium tetroxide for 2 h at 4 °C. The samples were dehydrated by passage through graded ethanol series and soaked in acetone mixture at room temperature. After embedding and polymerization, the samples were cut into ultra-thin sections of 60–80 nm. After 2% uranium acetate and 2.6% lead citrate double staining, electron microscopic images were observed using a transmission electron microscope (HITACHI, HT7800).

### TUNEL assay

TdT-mediated dUTP nick-end labeling (TUNEL) assay was used to quantify the number of pyroptotic cells. Pancreatic tissue Sect. (4 μm) were dewaxed and then stained for TUNEL assay following the manufacturer’s instructions (Beyotime, Shanghai, China). The staining of pyroptotic cells was observed using a fluorescence microscope. Five random fields were taken from each section to count the numbers of TUNEL-positive cells and the percentage of positive cells was calculated.

### Statistical analysis

Statistical analysis was performed using SPSS 24.0 software (SPSS, Inc., Chicago, IL, USA). Continuous variables conforming to normal distribution are presented as mean ± standard deviation (SD). Between-group differences were assessed using Student’s *t* test, while multi-group comparisons were performed using one-way analysis of variance (ANOVA) followed by post-hoc Tukey method. *P* values < 0.05 were considered indicative of statistical significance. Plots were generated with GraphPad Prism 8 software (San Diego, CA, USA).

## Results

### Silencing TRAF6 inhibited HTG-AP-induced pyroptosis in vitro

**Fig. 1:**
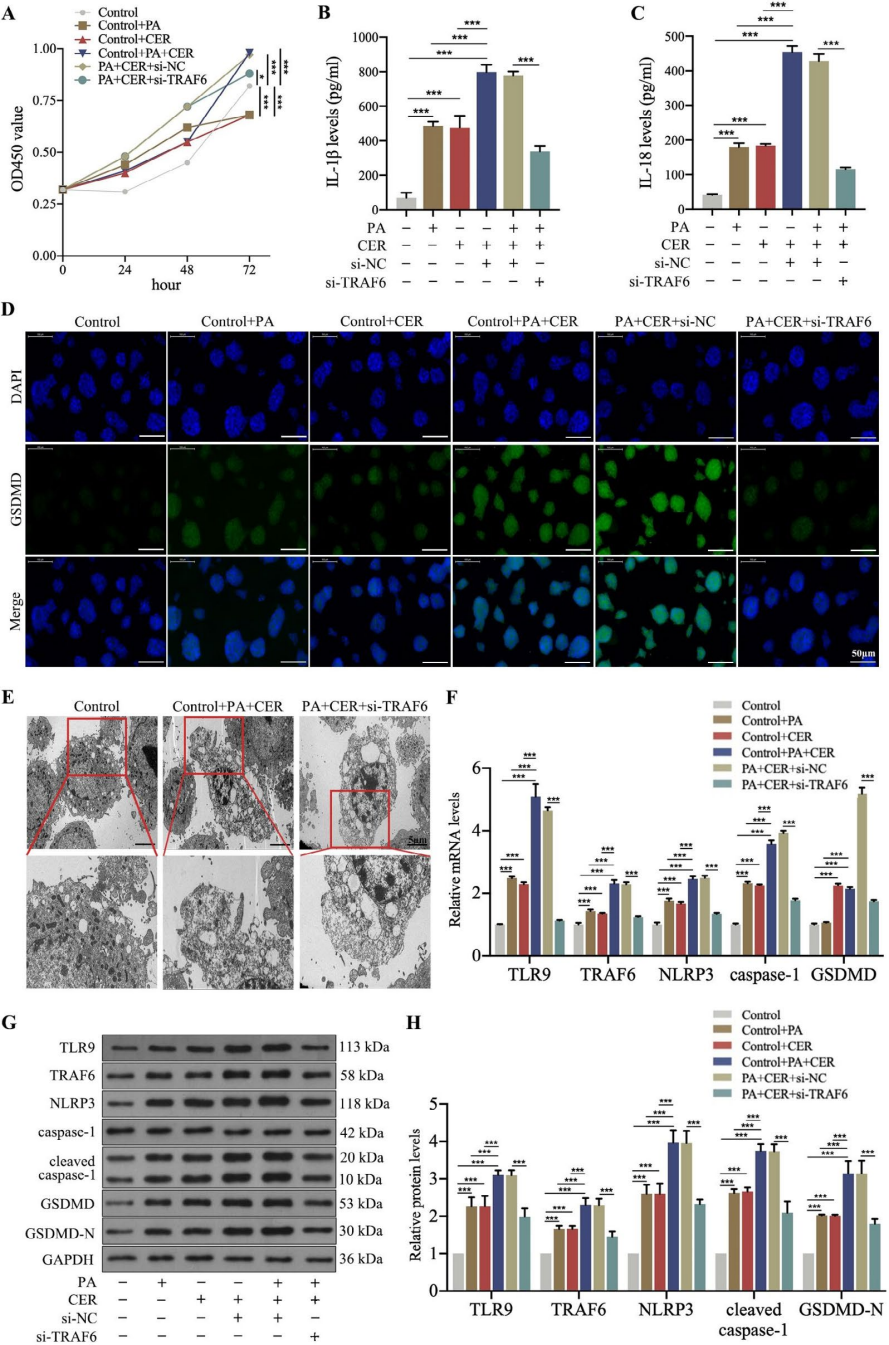
Silencing TRAF6 inhibited HTG-AP-induced pyroptosis in vitro. Rat pancreatic acinar AR42J cells were transfected with si-TRAF6 and then treated with 0.5 mmol/L palmitic acid and 5 nM cerulein for 24 h. **A)** The proliferative ability of cells was evaluated by CCK-8 assay. **B, C)** The levels of inflammatory mediators were measured by ELISA. **D)** The expression of GSDMD was detected by immunofluorescent staining (200×). **E)** Ultrastructure of pancreatic acinar cells was observed under transmission electron microscope. **F, G, H)** The levels of TLR9, TRAF6, NLRP3 and pyroptosis-related proteins were determined by RT-qPCR and Western blot. ^*^P < 0.05, ^**^P < 0.01 and ^***^P < 0.001

We first simulated HTG environment in vitro by treating rat pancreatic acinar AR42J cells with 0.5 mmol/L palmitic acid, and then examined whether treatment of HTG-AP cell models with 5 nM cerulein induces pyroptosis. After palmitic acid and cerulein treatment, the proliferative capability of the pancreatic acinar cells was decreased (Fig. 1A), and the levels of IL-1β and IL-18 as well as the expression of activated GSDMD were increased remarkably, mainly in pancreatic acinar cells (Fig. 1B, C, and D). On transmission electron microscopy, the morphological characteristics of pyroptosis (including nucleolysis, formation of intracellular vesicles, and cell membrane rupture) were found to be more obvious in the palmitic acid and cerulein-treated groups than in the control group (Fig. 1E). Results of RT-qPCR and Western blot assay showed that the expressions of TLR9, TRAF6, NLRP3, and pyroptosis-associated proteins-caspase-1 and GSDMD were elevated after treatment with palmitic acid and cerulein compared with the control group (Fig. 1F, G, and H). Importantly, inflammatory reaction and pyroptosis significantly increased after stimulation with palmitic acid plus cerulein.

To explore the consequences of TRAF6-pyroptosis interaction in HTG-AP, the expression of TRAF6 in pancreatic acinar AR42J cells was downregulated using siRNA transfection. We found that palmitic acid plus cerulein-stimulated si-TRAF6 pancreatic acinar cells showed increased viability, decreased inflammatory reaction, and decreased levels of activation as well as morphological changes of pyroptosis (Fig. 1A-H). The above results suggested that palmitic acid and cerulein treatment induced pyroptosis in pancreatic acinar cells and indicated the potential involvement of TRAF6 in pyroptosis in HTG-AP cell models.

### Inhibition of TRAF6 attenuated the severity of AP in rat models

**Fig. 2:**
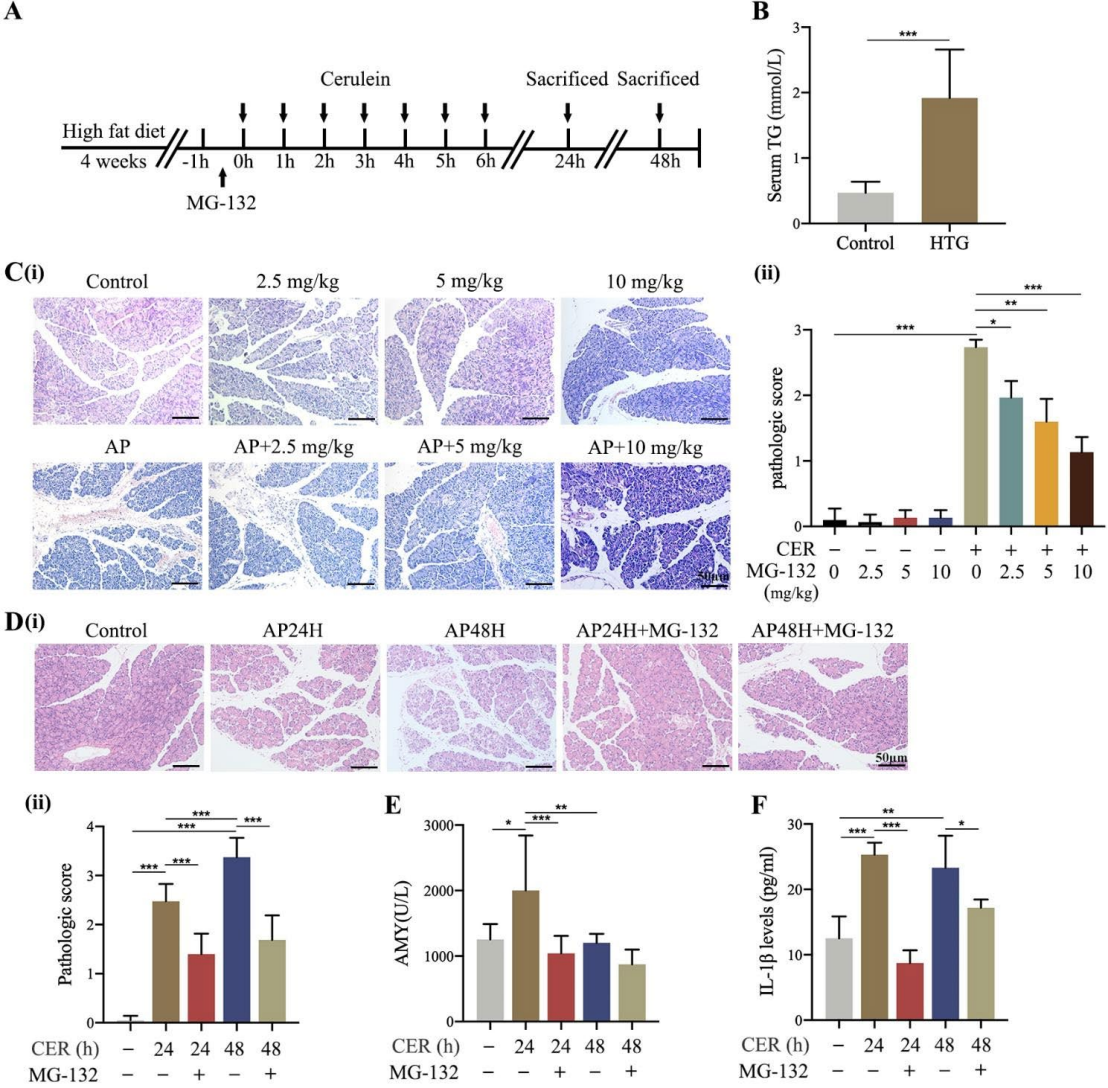
Inhibition of TRAF6 attenuated the severity of AP in rat models. **(A)** Schematic illustration of the process of establishment of HTG-AP rat model and administration of MG-132. **(B)** Serum TG levels in control and HTG rats were measured. **(C)** Different concentrations of MG-132 (2.5, 5, and 10 mg/kg) were injected intraperitoneally in rats 0.5 h before modeling. Rats were sacrificed at 24 h after the last injection of cerulein and the histopathologic score was assessed (HE staining 200×). **(D)** Rats were treated with 10 mg/kg MG-132 and sacrificed at 24 and 48 h. The histopathologic score was assessed (HE staining 200×). **(E)** Serum amylase levels in AP rats were measured. **(F)** Serum IL-1β levels in AP rats were measured by ELISA. ^*^P < 0.05, ^**^P < 0.01 and ^***^P < 0.001

For further investigating the relationship of TRAF6 and pyroptosis in HTG-AP rat models, MG-132 was used to inhibit the expression of TRAF6 and pyroptosis was also studied. The schematic illustration of the establishment of HTG-AP rat model and the administration of MG-132 is presented in Fig. 2A. High-fat diet rats had significantly higher serum TG levels compared to the control group (Fig. 2B). We first explored the optimal intervention concentration of MG-132 and examined whether inhibition of TRAF6 attenuates the severity of AP in rat models. Considering that the level of serum amylase does not accurately reflect the severity of AP, pathological changes were used to assess the pancreatic injury. Histopathologic examination confirmed that 10 mg/kg MG-132 had the most significant protective effect on AP rats (Fig. 2C). Compared with the control group, pancreatic tissue in the AP group showed widened interlobular spaces, interstitial edema, vacuolization, and inflammatory cell infiltration. In particular, a few necrotic pancreatic acinar cells were found in the AP48H group. Moreover, the corresponding histopathologic scores were significantly elevated, accompanied by increased serum levels of amylase and inflammatory cytokines. MG-132 treatment alleviated pancreatic damage, including histopathologic scores and serum levels of amylase and inflammatory cytokines (Fig. 2D, E, and F). These results showed that inhibiting TRAF6 was protective against AP in rats as evidenced by alleviated pancreatic injury and reduced inflammatory reaction.

### Inhibition of TRAF6 suppressed pyroptosis in AP rat models

**Fig. 3:**
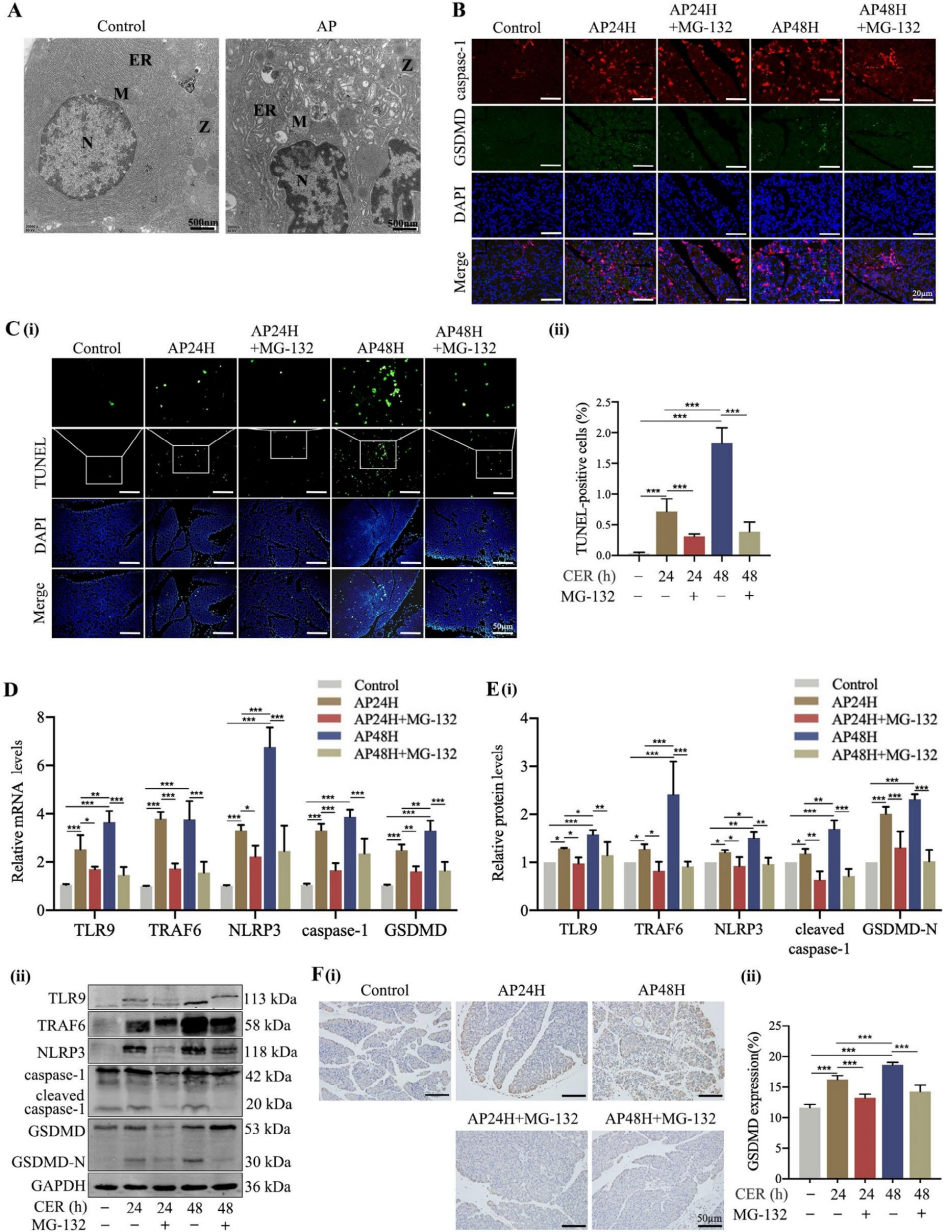
Inhibition of TRAF6 suppressed pyroptosis in AP rat models. **(A)** Ultrastructure of pancreatic acinar cells from AP rats was observed under transmission electron microscope; N: nuclear, M: mitochondrion, Z: zymogen granules, ER: Endoplasmic reticulum. **(B)** The expression of GSDMD in the pancreatic tissue of AP rats was detected by immunofluorescent staining (400×). **(C)** Pyroptotic cells in the pancreatic tissue of AP rats were detected by TUNEL staining (200×). **D, E)** The levels of TLR9, TRAF6, NLRP3, and pyroptosis-relaxed proteins in the pancreatic tissue of AP rats were determined by RT-qPCR and Western blot. **F)** The expression of GSDMD in pancreatic tissue of AP rats was detected by immunohistochemistry staining (200×). ^*^P < 0.05, ^**^P < 0.01 and ^***^P < 0.001

Transmission electron microscopy showed significant changes in the morphology of cerulein-injured pancreatic acinar cells compared to the control group. These changes included nuclear pyknosis, chromatin condensation and marginalization, mitochondrial swelling, and endoplasmic reticulum expansion (Fig. 3A). Immunofluorescent staining showed remarkably enhanced expression of pyroptosis-relaxed proteins caspase-1 and GSDMD in the pancreatic tissue compared to group control, especially in the AP48h group. MG-132 significantly inhibited pyroptosis in cerulein-induced AP rats, as shown by the reduction in red and green fluorescence, respectively representing caspase-1 and GSDMD proteins, at various time-points (Fig. 3B). These findings were consistent with results of TUNEL assay which showed reduced number of pyroptotic cells (Fig. 3C).

Thus, we further investigated the mechanism by which TRAF6 mediates pyroptosis in AP rat models. RT-qPCR and Western blot results showed decreased expressions of TLR9, TRAF6, NLRP3, and pyroptosis-relaxed proteins (including caspase-1 and GSDMD) after treatment with MG-132 (Fig. 3D, E). Furthermore, immunohistochemistry staining revealed even further amelioration of the expression of GSDMD (Fig. 3F). These results demonstrated that inhibition of TRAF6 suppressed pyroptosis and TLR9/TRAF6/NLRP3 signaling pathway activation in AP rats.

### Inhibition of TRAF6 attenuated the severity of HTG-AP in rat models

**Fig. 4:**
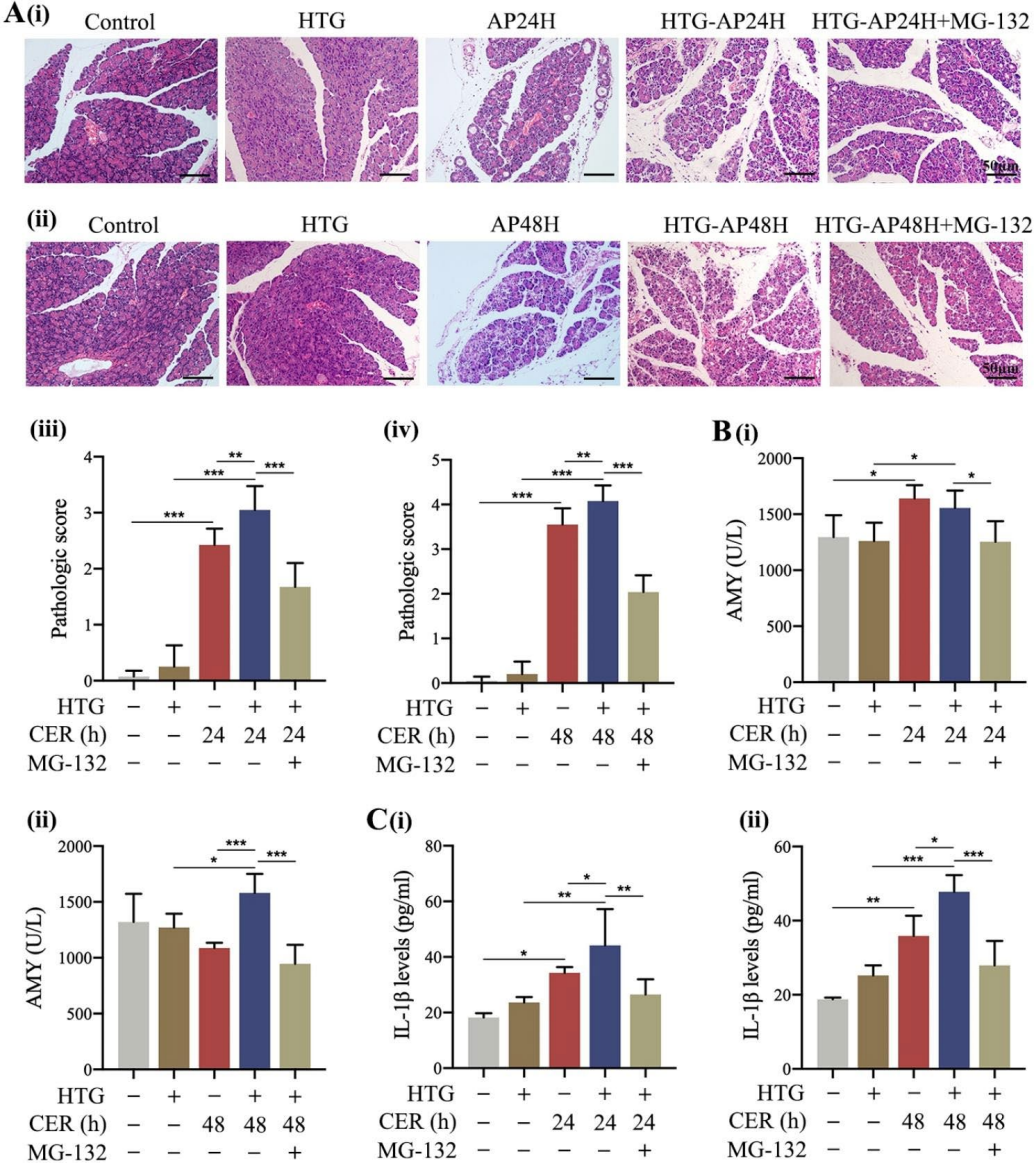
Inhibition of TRAF6 attenuated the severity of HTG-AP in rat models.**(A)** HTG rats were treated with 10 mg/kg MG-132 and sacrificed at 24 and 48 h. The histopathologic score was assessed (HE staining 200×). **(B)** Serum amylase levels in HTG-AP rats were measured. **(C)** Serum IL-1β levels in HTG-AP rats were measured by ELISA. ^*^P < 0.05, ^**^P < 0.01 and ^***^P < 0.001

Subsequently, we examined whether inhibition of TRAF6 attenuates the severity of HTG-AP in rat models. We first found that the severity of injury in pancreatic tissues from the HTG-AP group was markedly greater than that in the AP rats at 24 and 48 h, as evidenced by more necrotic pancreatic acinar cells (Fig. 4A). After MG-132 administration, the severity of pancreatic tissues was dramatically attenuated in HTG-AP + MG-132 group compared with that in HTG-AP group, including reduction of histopathologic scores as well as serum levels of amylase and inflammatory cytokines (Fig. 4A, B and C). These findings indicated that inhibition of TRAF6 attenuated the severity of HTG-AP in rat models.

### Inhibition of TRAF6 suppressed pyroptosis in HTG-AP rat models

**Fig. 5:**
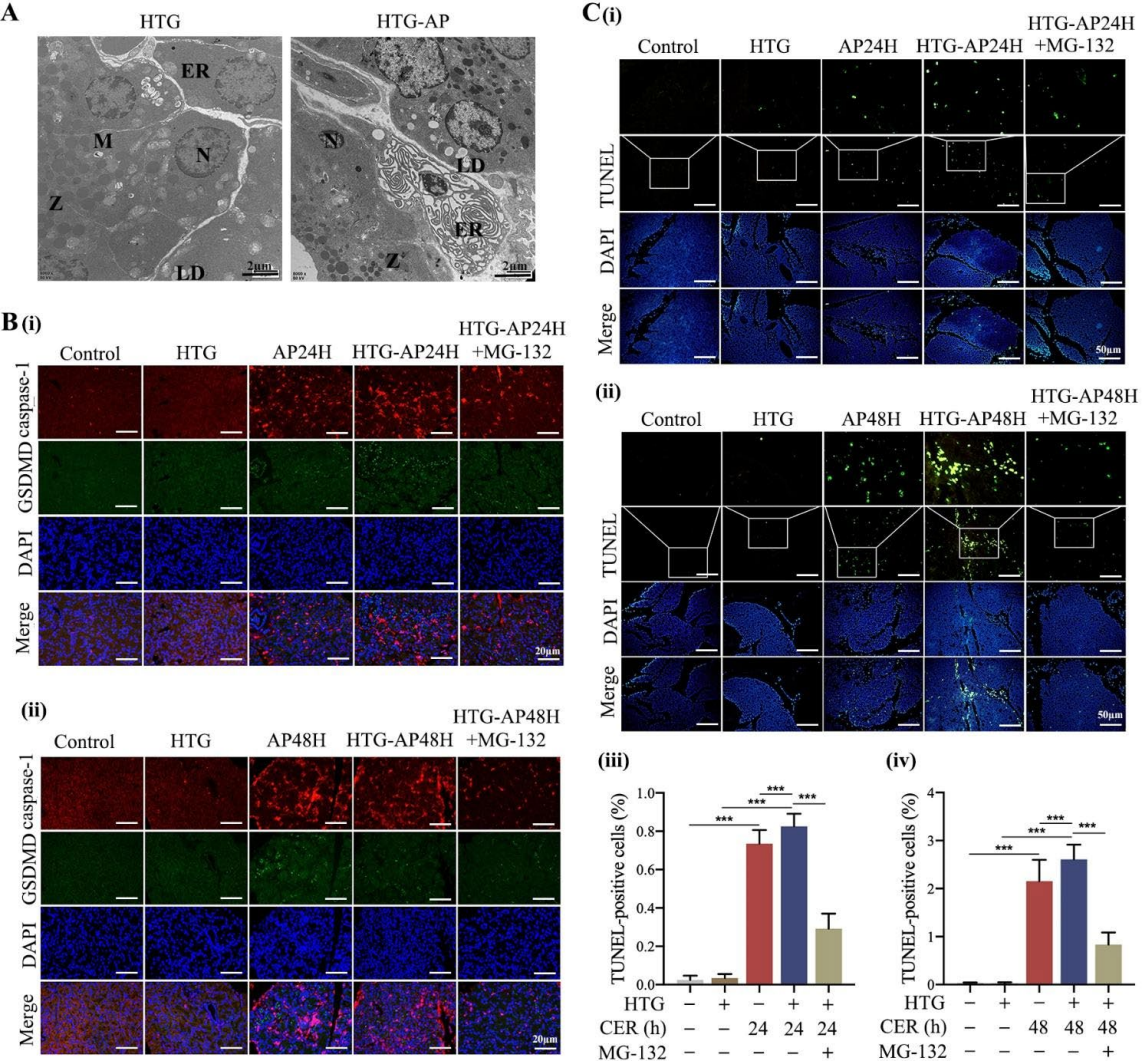
Inhibition of TRAF6 suppressed pyroptosis in HTG-AP rat models. **(A)** Ultrastructure of pancreatic acinar cells from HTG-AP rats was observed under transmission electron microscope; N: nuclear, M: mitochondrion, Z: zymogen granules, ER: Endoplasmic reticulum, LD: lipid droplet. **(B)** The expression of GSDMD in pancreatic tissue of HTG-AP rats was detected by immunofluorescent staining (400×). **(C)** Pyroptotic cells in pancreatic tissue of HTG-AP rats were detected by TUNEL staining (200×). ^*^P < 0.05, ^**^P < 0.01 and ^***^P < 0.001

Similarly, we investigated the presence of pyroptosis in HTG-AP rats. In addition to nuclear pyknosis, chromatin condensation and margination, and endoplasmic reticulum expansion, lipid droplets were also seen under a transmission electron microscope in HTG-AP rats (Fig. 5A). Compared with the HTG group, immunofluorescent and TUNEL staining showed greater activation of caspase-1 and GSDMD proteins and increased number of pyroptotic cells in the HTG-AP group (Fig. 5B, C). Of note, HTG-AP rats displayed more prominent pyroptosis compared with AP rats. Furthermore, similar positive effects of inhibiting TRAF6 were observed on immunofluorescent and TUNEL staining, which was consistent with reduced expressions of caspase-1 and GSDMD proteins and pyroptotic cells after MG-132 treatment in HTG-AP rats (Fig. 5B, C). Collectively, these findings demonstrated the occurrence of pyroptosis in HTG-AP rats, which may be associated with the regulation of TRAF6.

**Fig. 6:**
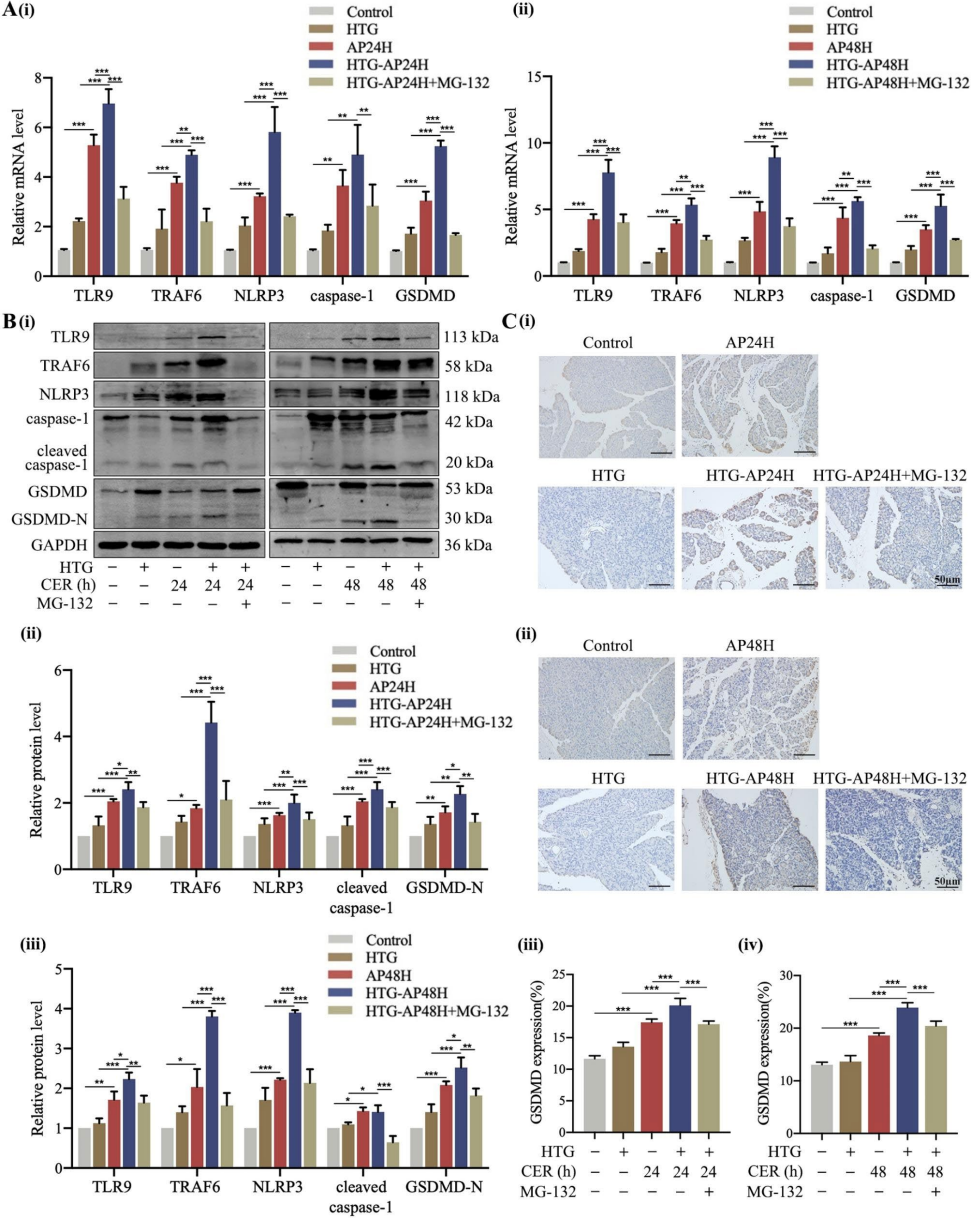
Inhibition of TRAF6 suppressed pyroptosis in HTG-AP rat models. **A, B)** The levels of TLR9, TRAF6, NLRP3, and pyroptosis-relaxed proteins in pancreatic tissue of HTG-AP rats were determined by RT-qPCR and Western blot. **C)** The expression of GSDMD in pancreatic tissue of HTG-AP rats was detected by immunohistochemistry staining (200×). ^*^P < 0.05, ^**^P < 0.01 and ^***^P < 0.001

Therefore, we next validated the mechanism by which TRAF6 mediates pyroptosis in HTG-AP rat models. MG-132 treatment greatly decreased the expressions of pancreatic TLR9, TRAF6, and pyroptosis-relaxed proteins in HTG-AP rats (Fig. 6A, B, and C). These findings indicated that inhibition of TRAF6 suppressed pyroptosis via TLR9/TRAF6/NLRP3 signaling pathway during HTG-AP.

## Discussion

HTG-AP has been widely studied because of its greater severity and the tendency for recurrence and rapidly progressive disease course [26]. HTG-AP is more likely to cause pancreatic necrosis than other forms of AP [27]. Enhanced inflammatory response is one of the main causes of the increased complications and mortality from HTG-AP [28]. Pyroptosis is an important innate immune defense mechanism against pathogens that entails excessive activation of inflammatory response leading to amplification of the inflammatory cascade [29]. The present in vitro and in vivo study confirmed the occurrence of pyroptosis in cerulein-induced AP and HTG-AP models. Specifically, the effects of AP and HTG-AP on pyroptosis were mediated via activation of inflammasome and release of inflammatory mediators. Of note, we observed more severe pancreatic necrosis, activation of pyroptosis, and inflammatory response in HTG-AP compared to AP. Furthermore, inhibition of TRAF6 markedly decreased the severity of AP and HTG-AP. This may be related to the inhibition of NLRP3-mediated pyroptosis, which is of great significance for the early treatment and prognosis of HTG-AP.

Clinically, Watts and Lloret Linares demonstrated that extreme HTG (serum TG > 20 mmol/L) is more likely to cause AP, suggesting that HTG may be an independent factor in the progression of AP [30, 31]. Pancreatic acinar cells are rich in lipase which can hydrolyze TG to produce free fatty acids (FFA). Accumulation of FFA triggers an inflammatory cascade through Ca^2+^ overload, microcirculation disorders, and release of inflammatory mediators, eventually leading to HTG-AP [32]. However, these pathogenic mechanisms have not been fully elucidated in the early severe development of HTG-AP. Pyroptosis is a kind of programmed cell death involving multiple complex signaling pathways. It has been shown to participate in the occurrence and development of various diseases [33–35]. Recent study has shown significant pyroptosis in in vitro and in vivo pancreatic injury models in a simulated HTG environment, indicating pyroptosis activation after FFA induction [36]. In addition to pancreatic injury, FFA-induced pyroptosis has also been reported in the context of cardiac and liver injury: inhibition of pyroptosis mitigated lipid metabolism disorders caused by obesity, as well as heart and liver damage [37, 38]. Notably, the development of feeding high fat diet in rodent models seems to consistently: obesity, HTG, and insulin resistance, similar to our study. Mechanically, there are three parts of FFA-activated pyroptosis including inflammasome formation, caspase-1 activation and release of IL-1β and IL-18 [39]. Thus, the primary aim of the present study was to investigate the differences in pancreatic damage, pyroptosis, and inflammatory response between cerulein-induced AP and HTG-AP. We observed that pyroptosis and the levels of inflammatory mediators were significantly increased after stimulation of pancreatic acinar cells with cerulein; this phenomenon was further enhanced after treatment with palmitic acid plus cerulein. Similarly, we found that HTG-AP contributed to more severe pancreatic damage in rats, and HTG was related to increased expression of NLRP3 and pyroptosis-related proteins and inflammatory response as well. Collectively, these findings point towards the potential participation of NLRP3-mediated pyroptosis in the early course of HTG-AP, which deserves further study.

There is sound evidence that genetic and pharmacologic inhibition of NLRP3 inflammasome activation and subsequent pyroptosis attenuates pancreatic acinar cell death, experimental pancreatic injury, and inflammatory reaction [40]. Previous study has found that activation of pyroptosis significantly aggravate AP, it might be associated with activating the NLRP3 inflammasome and promoting caspase-1 self-cleavage and maturation, accompany by release of IL-1β and IL-18 [41]. These studies indicate that the indispensable role of NLRP3 inflammasome activation in pyroptosis in AP has been confirmed. However, the upstream mechanism by which NLRP3-mediated pyroptosis affects AP remains unclear. An early study has demonstrated that knockout TLR9 and NLRP3 genes or intervention with TLR9 inhibitor reduced pancreatic edema and inflammatory response in AP [42]. TLRs are considered to be central receptors in innate immunity, while TLR/IL-1R domain is the core component for TLRs to transmit signals, such as TRAF6. Loss of TRAF6 has recently been reported to specifically inhibit TLR/IL-1R-mediated NLRP3 inflammasome activation [43]. Thus, it is plausible that regulating TRAF6 may affect NLRP3-mediated pyroptosis and excessive inflammatory reaction, and improve the prognosis of HTG-AP. Subsequently, we investigated whether regulating TRAF6 affects HTG-AP via the pyroptosis pathway, which would indicate the involvement of TRAF6 in the pathogenesis of HTG-AP. First, this finding is consistent with a previous study in which TRAF6 was found highly-expressed in both in vitro and in vivo AP and HTG-AP models, especially in HTG-AP groups [24]. Next, the *TRAF6* gene was knocked down by siRNA-transfection in pancreatic acinar cells according to the most significant knockout efficiency in previous study [44]. On the other hand, three doses of MG-132 (from low to high) were administered in the in vivo AP model, and the optimum dose to reduce pancreatic injury was found to be 10 mg/kg. The results suggest that MG-132, a TRAF6 inhibitor, improves pancreatic injury during AP. More specifically, genetic and pharmacological inhibition of TRAF6 in either cerulein-induced AP or HTG-AP inhibited pancreatic acinar cell death, accompanied by a decrease in the levels of inflammatory mediators and pyroptosis pathway-related mRNAs and proteins. Based on the above, our study demonstrated that inhibition of TRAF6 could restrain pyroptosis and the subsequent inflammatory response in HTG-AP.

A previous study showed a positive correlation between serum TG level and the severity of AP [45]. Our findings further indicated that HTG is an important factor leading to the aggravation of AP. We showed that TRAF6 may play a crucial role in the main mechanism of HTG-AP by regulating the NLRP3-mediated pyroptotic pathway. Further studies are required for in-depth characterization of the related mechanisms between TRAF6 and pyroptosis in the context of HTG-AP. However, a limitation of our study was that TRAF6 was not genetically knocked out in the in vivo model to verify its importance for pyroptosis in HTG-AP. Indeed, MG-132 is not a targeted inhibitor of TRAF6 and may accidentally affect pyroptosis through other signaling pathways in animal experiments. However, MG-132 is a protease inhibitor with wide-ranging cellular functions. Mechanically, MG-132 is involved in proteasome-targeted degradation of TRAF6 and IκBa, and regulates NF-κB activation[46]. It has also been reported to be able to inhibit TRAF6 via exogenous stimulation of TLRs interacting with IRAK-1 [47]. Additionally, studies have suggested a potential role of MG-132 in the treatment of pancreatic cancer, by reducing the expression of TRAF6 in tumor tissue and delaying tumor growth [48]. These findings indicate the involvement of MG-132 in reducing TRAF6 levels.

In summary, our findings indicate a vital role of pyroptosis in the early stage of HTG-AP. Regulation of TRAF6 was found to attenuate HTG-AP pancreatic injury, along with the alleviation of inflammatory response through the process of NLRP3-mediated pyroptosis. Pyroptosis, an early cellular event, is a potential therapeutic target for ameliorating the severity of HTG-AP. Moreover, TRAF6 is expected to be a powerful therapeutic strategy for future HTG-AP treatment.

## Data Availability

All datasets used and/or analyzed for this study are available from the corresponding author on reasonable request.
